# Using dietary exposure to determine sub-lethal effects from imidacloprid in two springtail (Collembola) species

**DOI:** 10.1007/s10646-023-02715-x

**Published:** 2023-11-21

**Authors:** Andreia Sofia Jorge Silva, Silje Marie Kristiansen, Sagnik Sengupta, Cornelis A. M. van Gestel, Hans Petter Leinaas, Katrine Borgå

**Affiliations:** 1 Section for Aquatic Biology and Toxicology, Department of Biosciences, University of Oslo, Oslo, Norway; 2 Department of Animal Biology, Faculty of Sciences, University of Lisbon, Lisbon, Portugal; 3 Amsterdam Institute for Life and Environment (A-LIFE), Faculty of Science, Vrije Universiteit, Amsterdam, The Netherlands

**Keywords:** Collembola, Neonicotinoids, Dietary exposure, Life history traits, Soil ecotoxicology

## Abstract

Standard toxicity tests expose springtails (Collembola) through soil, while dietary exposure tests with animals visible on a surface are less commonly applied. We refined a method for dietary chemical exposure for two widely distributed and abundant Collembola species: *Folsomia quadrioculata* and *Hypogastrura viatica* as existing methods were sub-optimal. Newly hatched Collembola were offered bark with a natural layer of Cyanobacteria that was either moistened with a solution of the neonicotinoid insecticide imidacloprid using a micropipette or soaked in the solution overnight. The first method was superior in producing a measured concentration close to the nominal (0.21 and 0.13 mg/kg dry bark, respectively), and resulting in sub-lethal effects as expected. The adult body size was reduced by 8% for both species, but egg production only in *H. viatica*. Contrastingly, soaked bark resulted in a measured concentration of 8 mg/kg dry bark, causing high mortality and no egg production in either species. Next, we identified the sub-lethal concentration-range by moistening the bark to expose *H. viatica* to 0, 0.01, 0.04, 0.13, 0.43 and 1.2 mg imidacloprid/kg dry bark. Only the highest concentration affected survival, causing a mortality of 77%. Imidacloprid reduced moulting rate and the body size at first reproduction. The age at first reproduction appeared delayed as some replicates did not reproduce within the experiment duration. The method of moistened bark for dietary exposure proved optimal to continuously study life history traits, such as growth and reproductive outcomes, which are important to understand effects on key events crucial for population viability and growth.

## Introduction

Globally, soils are increasingly impacted by insecticides. One of the most used insecticide groups worldwide are neonicotinoids (Jeschke et al. 2011), including imidacloprid. It was banned for outdoor agricultural use in Europe in 2018 (European Commission (2018)), but is still used in high volumes globally outside the EU (Chen et al. 2019). A large proportion of applied pesticide ends up in the soil, where it can remain for several years owing to high persistence (Goulson 2013). Field concentrations of imidacloprid (Silva et al. 2019) can cause negative effects for non-target, soil-living arthropods (de Lima e Silva. et al. 2017; van Gestel et al. 2017; Kristiansen et al. 2021). One such group is the Collembola (springtails), which are important members of the soil fauna (Rusek 1998), and among the soil animals that are most sensitive to pesticides originating from agriculture (Joimel et al. 2022), such as neonicotinoids.

Collembola are fairly small in size (usually 0.5–2 mm), but with high abundance. By affecting decomposition processes they contribute to the plant nutrient turnover (Petersen and Luxton 1982; Birkemoe and Liengen 2000). Because of their ecological role, it is important to understand the impacts of contaminants on the fitness (vital rates) of Collembola. To improve our insight in processes underlying such impacts, we focus on how to study sub-lethal effects on life history traits, such as growth and egg production. In most terrestrial systems, the collembolan species diversity is high, with considerable site-specificity, which makes it important to study multiple species (Hertzberg and Leinaas 1998; Konestabo et al. 2022). Stress, e.g. induced by pesticide exposure, can lead the Collembola to allocate and prioritise energy to specific life history traits to achieve or maintain homeostasis (Stearns 1989; Stam et al. 1996; Hertzberg and Leinaas 1998; Choi et al. 2008). Thus, with energy restriction caused by contaminant stress, Collembola´s moulting process might be negatively affected (Lee et al. 2019; Zhang and Qiao 2020), consequently reducing their growth rate, and possibly delaying maturation. In another arthropod (brown shrimp, *Farfantepenaeus aztecus*), imidacloprid has shown to decrease growth (Al-Badran et al. 2019), but this is not yet studied for Collembola. Ultimately, reduced growth and delayed or reduced reproduction affect the overall fitness and population growth.

Traditional toxicity tests with soil animals are designed to administer the toxicant directly into the soil (International Organization for Standardization, 2011; International Organization for Standardization, 2014; OECD 2009). These standardised tests are important tools in the risk assessment of soil contaminants, allowing the assessment of concentration levels for ecological risks, and with high reproducibility allow direct comparison between effects from different contaminants on representative soil organisms. They involve exposure both via soil water and food, but allow documentation of effects only after the surviving animals have been extracted from the soil. However, to study impacts on life history traits, much more frequent observations are needed. This requires that animals can be observed active on a surface over time for monitoring effects throughout the experimental period. Such continuous observation may be done on a surface of compressed soil (Campiche et al. 2006; Jensen et al. 2001; Lee et al. 2018), or plaster of paris, while the animals are exposed through the diet. Previous ecotoxicological studies using dietary exposure most commonly spiked their exposure medium through soaking it in a solution, using yeast (e.g., Bruus Pedersen et al. 2000; Fountain and Hopkin 2001), algae (e.g., van Straalen et al. 1987; Hensbergen et al. 2000), or Cyanobacteria scraped off bark (Sengupta et al. 2021; Kristiansen et al. 2021) as diet. Another less common spiking method is by moistening the feed with droplets of the spiking solution, as done when exposing isopods through leaves (Tourinho et al. 2015). To assess the exposure after soaking the diet, the toxicant concentration in the diet needs to be analysed, while nominal concentrations can also be calculated when spiking through moistening. To our knowledge there are no studies comparing these two ways of spiking.

Collembola differ in vertical distribution from surface activity to different soil depths (Hopkin 1997). Their habitat variation affects many aspects of their life adaptation as well as exposure and sensitivity to pesticides (Fountain and Hopkin 2004; Konestabo et al. 2022). Even though there are more than 9400 Collembola species described (Bellinger et al. 1996–2022), the effects of toxic exposure are only studied using a few, but with indications of differences in species sensitivity (e.g. Lee et al. 2020; Ferreira et al. 2022). In the present study, we target *Folsomia quadrioculata* (Tullberg, 1871) and *Hypogastrura viatica* (Tullberg, 1872), which both have wide geographical distribution in the northern hemisphere (Convey et al. 1999; Jensen et al. 2006). *F. quadrioculata* is a soil litter-dwelling Collembola, while *H. viatica* is surface-dwelling and more mobile (Hertzberg et al. 2000; Krab et al. 2010). These two species can thus be considered good candidate model species for comparing sensitivity to pesticide exposure, as they are likely to differ in response based on their adaptations to different microhabitats. Previous studies indicate that *H. viatica* is highly sensitive to imidacloprid (Kristiansen et al. 2021), while *F. quadrioculata* can tolerate relatively higher concentrations (Sengupta et al. 2021). However, both these studies applied a method of soaked scraped Cyanobacteria on filter, which was sub-optimal for *H. viatica*, by reducing reproduction in control animals (Kristiansen et al. 2021). In *F. quadrioculata*, the use of scraped Cyanobacteria made handling of juveniles challenging for large-scale comparisons involving multiple populations and treatments (Sengupta, S., personal comment). The dietary exposure method therefore needed improvement. A direct comparison of the sensitivity between the two species to contaminant exposure has also not yet been made.

The aim of this study was to determine the sub-lethal effects of pesticide exposure in Collembola, by exposing them through their diet, while visible on a surface. First, we compared two methods of spiking feed with imidacloprid, and tested its validity on two species, hypothesising that *F. quadrioculata* would be less sensitive to imidacloprid than *H. viatica*. Second, to confirm suitability of the method considered most reliable in the first experiment, we performed a concentration-response experiment on *H. viatica*. Here, we identify the concentration range of imidacloprid optimal to study sub-lethal responses. Only *H. viatica* was used in this second part of the study, as their moulting rate, linked to growth, is easier to document in this species than in *F. quadrioculata*. We determined survival, age and size at first reproduction, as well as moulting rate, and expected the range of imidacloprid concentrations to affect the reproductive traits and growth negatively, while not increasing mortality.

## Materials and methods

### Test species

Both test species were collected from Svalbard, Norwegian Arctic; the soil litter-dwelling (hemi-edaphic) *F. quadrioculata* from Little State Island (81°N, 20°E), and the surface-dwelling (edaphic) *H. viatica* from the bay Fjortende Julibukta (79°N, 12°E). The climate on Svalbard is in general cold, but the Collembola are only active during the summer season. In July 2022, the temperature measured at Ny-Ålesund weather station ranged from 2.5 to 16 °C (Yr 2022). The Collembola were cultured for several years at the University of Oslo and should thus not have any parental or environmental effects. The cultures were kept in cylindrical containers with a radius of 1.5 cm and a height of 3.5 cm, with a 0.5–1.0 cm high layer of moistened plaster, darkened with a little activated charcoal. Once a week, a few drops of distilled water were added to maintain the optimal moisture level in the boxes. The cultured Collembola were fed weekly with small pieces of dry bark covered with a layer of Cyanobacteria. Branches were gathered from local Littleleaf Linden (*Tilia cordata*) trees, usually after strong winds in which dead branches were broken off from the tree. The branches were defaunated overnight at −80 °C and left to dry at room temperature. When dry, it is easy to peel off the outer layer of the bark without the need for any equipment, ensuring an equal thickness of the bark. The bark has a uniform layer of Cyanobacteria, with a black, crust-like appearance (Supplementary Fig. S1 in Supplementary Information). The Collembola only eat the black crust, and not the bark itself. All cultures were kept in incubators at a temperature of 15 °C.

### Comparison of spiking methods

We found the need to develop an additional method for dietary exposure, because our focal species do not reproduce at a normal rate when fed baker´s yeast (H. P. Leinaas, personal observation), which is a common food source for Collembola in the laboratory. Feeding with unicellular green algae, which is another common food source, was also problematic, as such aquatic algae require filtering, which interfered with a previous dietary exposure experiment (comment in Supplementary Information, section S1). For our local area, the quality of green algae from bark was highly variable between trees and over time within trees, because the succession of the green algal community. By far, the best breeding results were obtained by feeding the animals with bark covered with a natural layer of Cyanobacteria. We therefore decided to further develop a previous applied method using Cyanobacteria that was scraped off the bark using a scalpel and filtered to obtain a homogenous “powder” (Sengupta et al. 2021; Kristiansen et al. 2021). In the current study we used intact pieces of bark with the natural layer of Cyanobacteria. Imidacloprid (CAS 138261-41-3, Sigma-Aldrich, St. Louis, USA, 99.7% purity) was dissolved in distilled water to obtain a solution concentration of 129 µg/L, a concentration that we expected to be sub-lethal to *H. viatica* based on our previous experiment (Kristiansen et al. 2021). Thin pieces of defaunated dried bark with a natural uniform layer of Cyanobacteria (hereafter referred to as bark for simplicity) were randomly divided into five containers, to be prepared for five treatments, including three controls:

(I) Dry bark (control): unprocessed;
(II) Moistened bark (control): bark was cut into pieces of 25 mg and moistened with 25 µL distilled water;
(III) Moistened spiked bark: bark was cut into pieces of 25 mg and moistened with 25 µL imidacloprid solution;
(IV) Soaked bark (control): bark was soaked overnight in distilled water; or
(V) Soaked spiked bark: bark was soaked overnight in the imidacloprid solution.

After preparation, the bark was dried and stored in the dark at room temperature to avoid degradation of imidacloprid. Bark was offered to the Collembola in a size that ensured ad libitum food accessibility according to their life stage, i.e., the spiked 25 mg bark pieces were cut into smaller pieces to be offered in the early phases of the test and bark size offered was increased with age.

### Study design of experiment 1: establishing the method

**Fig. 1:**
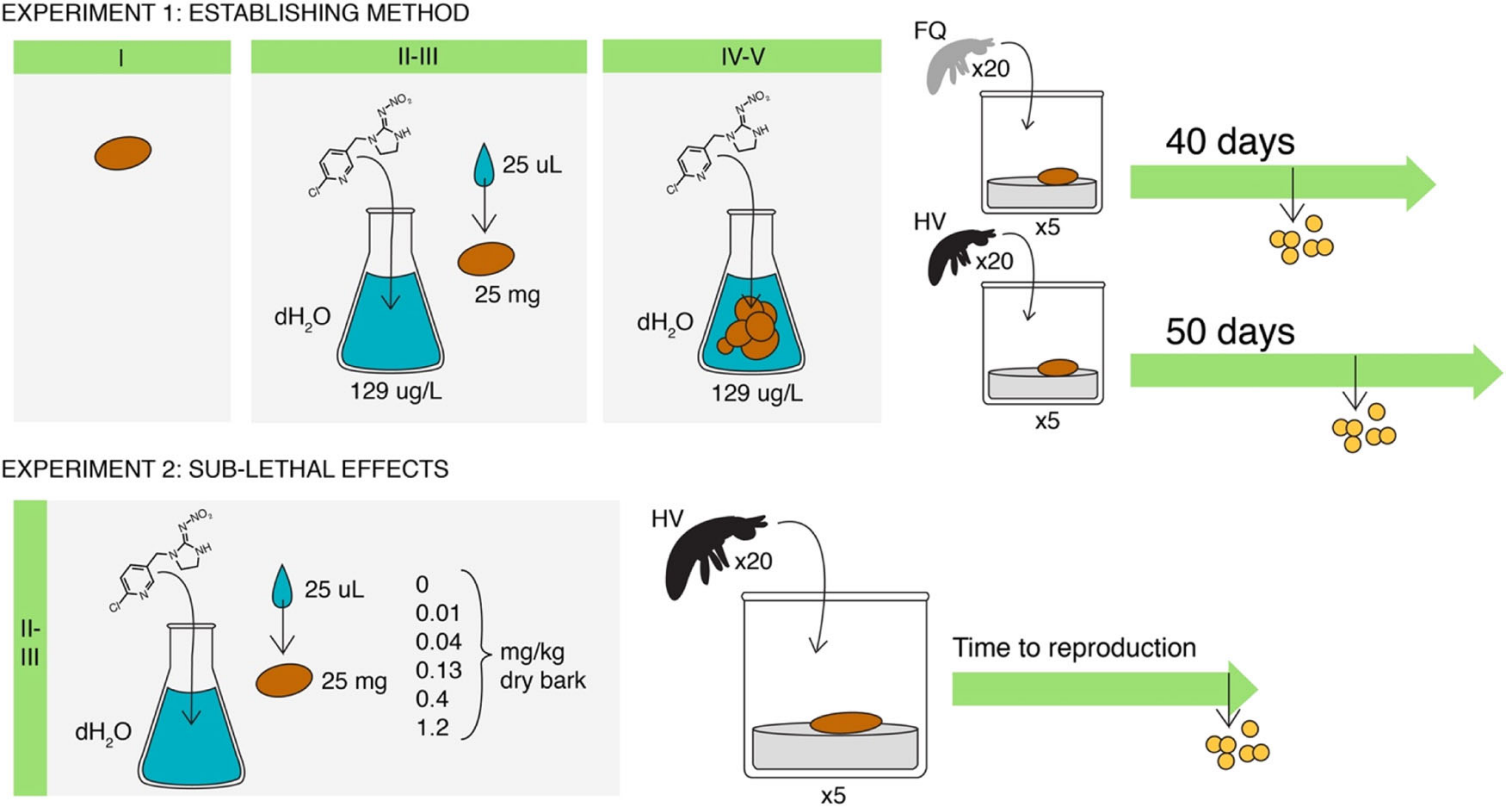
Experimental set up of two experiments, of which the first was conducted to establish a suitable spiking method of bark for dietary exposure of Collembola to imidacloprid, and the second confirmed a concentration range suitable for studying sub-lethal effects. Treatments included: I: dry bark (control), II: moistened bark (control), III: moistened bark spiked with imidacloprid, IV: soaked bark (control), V: soaked bark spiked with imidacloprid. Abbreviations: FQ *Folsomia quadrioculata*, HV *Hypogastrura viatica*

Five replicates of *F. quadrioculata* and *H. viatica* were exposed to treatment I-V at 20 °C (Fig. 1). This temperature is within the temperature range for both species´ physiological tolerances, i.e., 20 °C is not a stressor. Each replicate consisted of approximately 20 newly hatched juveniles (<24 h old), kept in similar containers as described for the cultures under “Test species”. The experimental period was 40 days for *F. quadrioculata* and 50 days for *H. viatica*, allowing approximately 2 weeks of egg production after their expected age at first reproduction at 20 °C (FQ: 25 days, Sengupta et al. 2017; HV: 35 days, unpublished data). Every three days, eggs and dead animals were counted and removed, and the bark was replaced to avoid fungal growth and to maintain the imidacloprid exposure level. At experiment termination, the Collembola were harvested in 70% ethanol, followed by heating to approximately 70 °C, to stretch the animals. The animals were photographed, and their individual body lengths were measured using the image software Leica Application Suite 4.5 software.

### Study design of experiment 2: sub-lethal concentration range

To establish a concentration range suitable for studying sub-lethal effects, we exposed *H. viatica* using the superior method from the first experiment, spiking the bark by moistening (treatment II-III, Fig. 1). Defaunated, dried bark was removed from the branches in thin layers, cut into pieces of 25 mg, and assigned randomly to 6 containers, one for each treatment. Each piece was moistened with 25 µL imidacloprid solution, giving the following nominal concentrations: 0.01, 0.04, 0.13, 0.4, and 1.2 mg/kg dry bark (a factor of 3 between concentrations, rounded digits). For the control, bark was moistened with distilled water. Each concentration had five replicates, consisting of approximately 20 newly hatched juveniles (<24 h old), kept in containers described for the cultures. Every three days throughout the experiment, the bark was replaced, animals and shed exuvia were counted, and dead animals removed. The experiment was conducted at 20 °C, and it lasted until the animals first reproduced, i.e., each replicate was harvested in ethanol on the day the first eggs were observed. The harvested animals were heated to stretch and body size measured as described for experiment 1. Two replicates that did not reproduce within the duration of the experiment were harvested at 1.5 times the age at first reproduction of cultures at 20 °C (unpublished data), i.e., at age 53 days. As four control boxes were erroneously placed at 15 °C for one day, rather than 20 °C, four additional experimental boxes were included at 20 °C to eliminate any potential temperature effect, i.e., we had nine experimental boxes for the control.

### Chemical analysis of bark

Approximately 1 g bark from treatment II-V in experiment 1, and all treatments in experiment 2 was homogenised separately using a hand mixer and divided into three replicates for quantification of imidacloprid exposure concentrations at the Institute for Water Research (NIVA) in Oslo, as described by Sengupta et al. (2021). Imidacloprid was extracted from the bark samples with acetonitrile (MeCN) and analysed using high-performance liquid chromatography-mass spectroscopy analysis. The detection limit (LOD) was 0.0001 mg/kg imidacloprid.

### Data treatment and statistical analyses

All statistical analyses and graphics were performed with RStudio (R Core Team 2019), with the significance level of 5%. Data was checked for assumptions of normality and homoscedasticity using Shapiro Wilk’s and the Levene’s tests. If the assumptions were not met, data was square root or log transformed, and if the assumptions were still not met, non-parametric tests were used as indicated.

#### Experiment 1

The age at first reproduction (in days) was defined as the average age between the observation where the first batch of eggs were found and the previous observation. Egg production was presented as cumulative number of eggs per animal. The survival was analysed with Kaplan-Meier statistics using the R-package *survival* (Therneau and Grambsch 2000), in which we censored lost animals (see comment in Supplementary Section S2). A log-rank test was used to compare survival curves. The sub-lethal endpoints were analysed using analysis of variance (ANOVA), followed by Tukey’s honestly significant difference test. Age at first reproduction was tested for differences only for *F. quadrioculata*, due to limited number of data points available for *H. viatica*.

#### Experiment 2

For all endpoints, there was no difference between the four control boxes with a one-day misplacement at 15 °C and the five experimental boxes placed continuously at 20 °C, i.e., control had nine replicates compared to five for the other treatments. The age at first reproduction was defined as in experiment 1. For two experimental boxes that did not reproduce within the duration of the experiment, we estimated an age at first reproduction of 70 days, which is twice the age at first reproduction for this population at 20 °C (mean = 35 days, SD = 3.5, min = 33, max = 45, n = 10, unpublished data). One outlier from the control was removed (63 days). The moulting rate was calculated by dividing the number of shed exuvia per observation by the number of animals at each time point, summarising the cumulative number of moults per animal and dividing it on their age in days. Body size at first reproduction was defined by the median for the experimental box. The survival was analysed in a similar manner as for experiment 1. The effects of imidacloprid on sub-lethal endpoints were analysed with ANOVA, followed by Tukey´s HSD test, which also identified the No Observed Effect Concentration (NOEC) and the Lowest Observed Effect Concentration (LOEC) based on the exposure concentrations. Due to unequal variance, also after transformation, body size at first reproduction was analysed with a Kruskal-Wallis rank sum test, followed by a Pairwise Wilcox test. The relationships between sub-lethal endpoints were analysed using linear mixed-effect models, with experimental box-id as random factor. The analysis of body size and moulting rate included replicates where no reproduction was documented.

## Results

### Experiment 1 – chemical analysis of feed

The measured concentration of imidacloprid in moistened spiked bark was 1.6 times higher than the nominal concentration, whereas the measured concentration in the soaked bark was 38 times higher than the measured moistened spiked bark (the nominal concentration could not be calculated) (Table 1). Low concentrations of imidacloprid were found in the control bark samples, close to the detection limit of 0.0001 mg/kg dry bark.

**Table 1:** Imidacloprid concentrations in bark treated with distilled water (control) or imidacloprid solution, either by moistening the bark with a droplet of imidacloprid solution, or soaking the bark in an imidacloprid solution overnight

(No.) Treatment	Nominal concentration (mg/kg)	Measured concentration (mg/kg)
(II) Moistened bark (control)	0	0.0003
(III) Moistened spiked bark	0.13	0.21
(IV) Soaked bark (control)	0	0.0014
(V) Soaked spiked bark	NA^a^	8.0

^a^*NA* not available, as it cannot be calculated

### Experiment 1 – survival and sub-lethal responses to imidacloprid

**Fig. 2:**
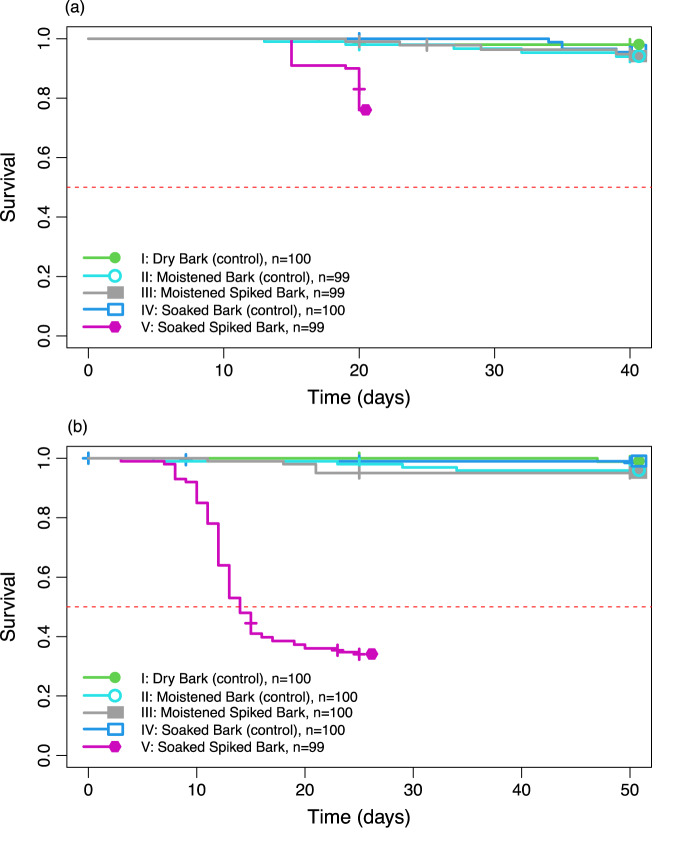
Proportions of survival for (**a**) *Folsomia quadrioculata* and (**b**) *Hypogastrura viatica* (Collembola) dietary exposed to imidacloprid through bark comparing two spiking methods: soaked in an imidacloprid solution overnight versus moistened with a droplet of imidacloprid solution using a micro-pipette. The spiked treatments had measured imidacloprid concentrations of 0.21 mg/kg and 8.0 mg/kg for the moistened and soaked spiking method, respectively. The red line marks the survival proportion of 0.5

Both species had high survival (>90%) in all three control treatments, thus meeting the quality assurance criteria of 80% given in standardised toxicity tests (OECD 2009). Exposure to imidacloprid in moistened bark was sub-lethal for both species, with a high mean survival of more than 90% for both species (Fig. 2). Exposure to imidacloprid in soaked bark resulted in reduced survival (Fig. 2, log-rank test: *p* < 0.0001 for both species), with survival being 76 and 35% for *F. quadrioculata* and *H. viatica*, respectively.

**Fig. 3:**
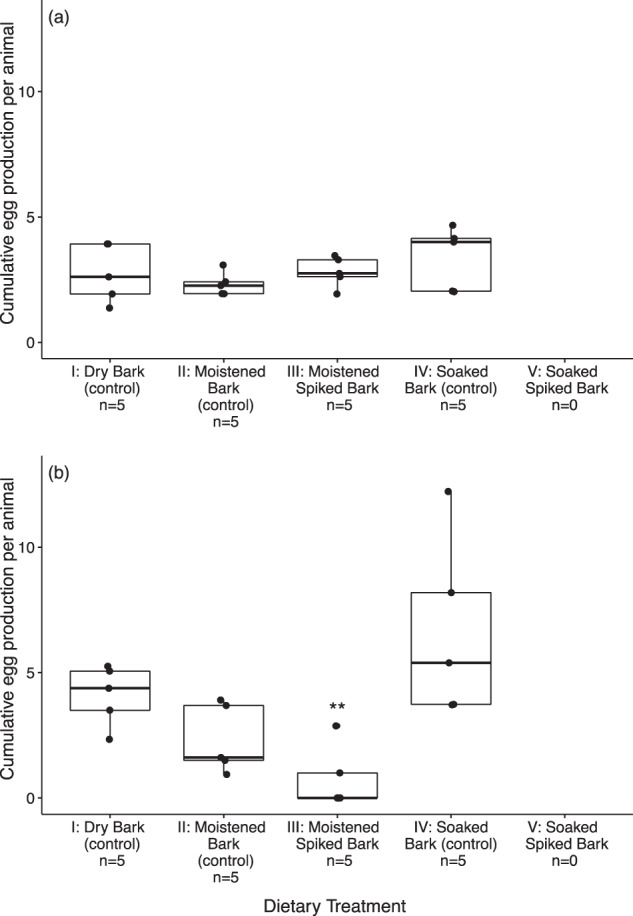
Cumulative egg production by (**a**) *Folsomia quadrioculata* and (**b**) *Hypogastrura viatica* (Collembola) dietary exposed to imidacloprid through bark comparing two spiking methods: soaked in an imidacloprid solution overnight versus moistened with a droplet of imidacloprid solution using a micro-pipette. The spiked treatments had measured imidacloprid concentration of 0.21 mg/kg and 8.0 mg/kg for the moistened and soaked spiking method, respectively. Bark soaked with imidacloprid caused high mortality and no reproduction, hence n = 0. Data presented as median, quartiles and 10–90 percentiles. (**) represents significant level *p* < 0.001 in comparison with the controls (Tukey’s HSD)

Both species reproduced in all control replicates. The mean age at first reproduction for *F. quadrioculata* exposed to imidacloprid in moistened bark was not different from the controls (ANOVA, *p* = 0.201, *F* = 1.728) (Supplementary Fig. S2, Supplementary information), and neither was its egg production (Fig. 3a, ANOVA, *p* = 0.513, *F* = 0.798). There was no egg production in *F. quadrioculata* exposed to imidacloprid in soaked bark (Fig. 3a). *H. viatica* only had egg production in two replicates exposed to imidacloprid in moistened bark within the experiment duration of 50 days (Supplementary Fig. S2, Supplementary Information), and it was thus not possible to conduct statistical analysis on age at first reproduction. Imidacloprid in moistened bark reduced mean egg production in *H. viatica* by 65 – 88% compared to the controls (Fig. 3b), although not significantly different from the moistened bark control (Tukey’s HSD: *p* > 0.05). No difference was found between the three controls (Tukey’s HSD: *p* > 0.05). There was no egg production in *H. viatica* exposed to imidacloprid in soaked bark (Fig. 3b).

Exposure to imidacloprid in moistened bark reduced the body size at age 40 days for *F. quadrioculata* and age 50 days for *H. viatica* by 8.3 and 7.7%, respectively, compared to their respective moistened bark control (Supplementary Fig. S3, Supplementary Information*, F. quadrioculata:* Tukey´s HSD, *p* < 0.001; *H. viatica:* Tukey´s HSD, *p* < 0.03). No difference was found in body size between the three controls for the respective species (Tukey´s HSD, *p* > 0.05 for all six combinations). We did not measure body size of animals exposed to soaked bark as all individuals died or were lost.

### Experiment 2 – chemical analysis of feed

The chemical analyses of imidacloprid confirmed that bark spiked by moistening had concentrations within 20% of the nominal ones, and with low variation among replicates (Table 2).

**Table 2:** Imidacloprid concentrations in bark spiked either by moistening with a droplet of imidacloprid solution and used in the sub-lethal exposure experiment with *Hypogastrura viatica* (Collembola)

Nominal concentration (mg/kg dry bark)	Mean measured concentration, *n* = 3 (mg/kg dry bark)	Standard Deviation, *n* = 3 (mg/kg dry bark)	Difference between measured and nominal concentrations (%)
0	0.0005	0.0004	–
0.01	0.01	0.0009	4
0.04	0.04	0.004	5
0.13	0.11	0.019	13
0.40	0.40	0.028	8
1.2	1.0	0.06	18

### Experiment 2 – responses to imidacloprid

**Fig. 4:**
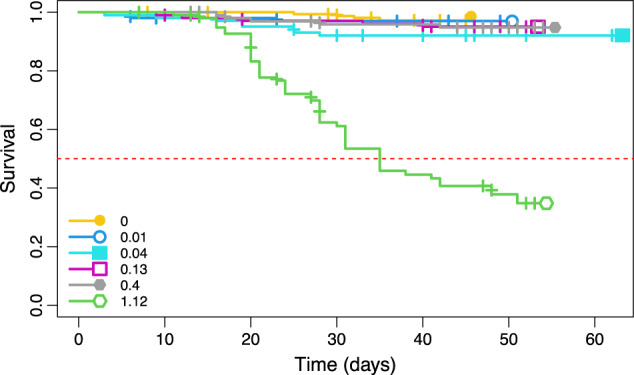
Proportions of survival of Hypogastrura viatica (Collembola) dietary exposed to imidacloprid (mg/kg dry bark). The red line marks the survival proportion of 0.5

The four lowest imidacloprid concentrations were in the sub-lethal range for *H. viatica*, with survival above 90% for all treatments, while the highest concentration of 1.2 mg/kg dry bark reduced survival to 33% (Fig. 4; log-rank test: *p* < 0.0001).

**Fig. 5:**
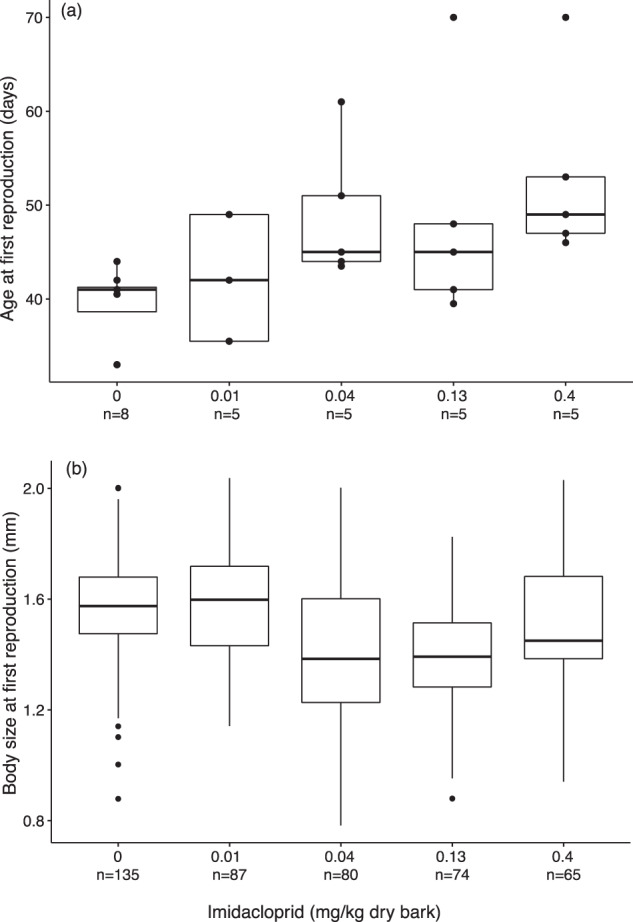
Age (**a**) and body size (**b**) at first reproduction for *Hypogastrura viatica* (Collembola) dietary exposed to imidacloprid. For the determination of the age at first reproduction, the two outlier values at 0.13 and 0.4 mg/kg dry bark were estimated, as these replicates did not reproduce within the duration of the experiment. Data points were set to 10 days after the replicate with the highest age for first reproduction, which was 60 days; 70 days is twice the mean reproductive age of H. viatica cultures at 20 °C. Data presented as median, quartiles, 10–90 percentiles and outliers

Due to high mortality and no reproduction in *H. viatica* exposed to the highest concentration of imidacloprid, the 1.2 mg/kg dry bark treatment was excluded from the analyses of the reproductive endpoints. The age at first reproduction was not affected by imidacloprid, except for the highest sub-lethal concentration of 0.4 mg/kg dry bark (Tukey HSD: *p*-value = 0.05), i.e., the LOEC, which had a mean increase of 13 days compared to the control (Fig. 5a). Reproduction was observed in all five replicates exposed to either of the two lowest imidacloprid concentrations, 0.01 and 0.04 mg/kg dry bark. At 0.13 and 0.4 mg/kg dry bark, four out of five replicates reproduced within the experiment duration, but as age at first reproduction at 0.13 mg/kg dry bark was not significantly different from the control (Tukey HSD: *p*-value = 0.32), this is the NOEC for age at first reproduction. There is some uncertainty linked to the age at first reproduction determined for 0.13 and 0.4 mg/kg dry bark, as the two replicates with no egg production within termination were given an estimated value for their age at first reproduction adding 10 days to the highest documented age at first reproduction.

Body size at first reproduction was reduced by imidacloprid at all sub-lethal concentrations (Pairwise Wilcox test: *p* < 0.001), except for the lowest of 0.013 mg/kg dry bark (Pairwise Wilcox test: *p* = 0.611), i.e., the NOEC for body size at first reproduction (Fig. 5b). The animals exposed to 0.04 mg/kg dry bark, i.e., the LOEC, had a reduction of 10.8% in median body size at first reproduction compared to the control. Exposure to the higher concentrations of 0.13 and 0.4 mg/kg dry bark reduced the median body size with 12.6 and 8.2%, respectively. Body size at first reproduction was dependent on imidacloprid concentration (linear mixed-effect model, *p* = 0.016), but there was no consistent trend in the relationship between age and body size at first reproduction within the imidacloprid treatments (Supplementary Fig. S4, Supplementary Information).

The moulting rate (number of shed exuvia per animal per day) was unaffected by exposure to imidacloprid at 0.01, 0.04, 0.13 and 0.4 mg/kg dry bark (Tukey´s HSD, *p* = 0.1; 0.17; 0.54; 0.89, respectively, Supplementary Fig. S5, Supplementary Information). Only animals exposed to the highest and lethal imidacloprid concentration of 1.2 mg/kg dry bark, had a reduced moulting rate of 78% compared to control (Tukey´s HSD: *p* = 0.05). The NOEC and LOEC for moulting rate were thus 0.4 and 1.2 mg/kg dry bark, respectively. The body size of *H. viatica* increased with an increasing moulting rate (linear mixed-effect model, slope = 3.43; *p* = 0.0001), of which the latter is a proxy for growth, and thus a method of quality assurance. The positive relationship was independent of exposure concentration (linear mixed-effect model, *p* = 0.7, Supplementary Fig. S6, Supplementary Information).

## Discussion

In the present study we have established that spiking bark by moistening provides predictable concentrations, and is thus an optimal method for exposing two ecological relevant Collembola species with adaptations to different habitats, allowing direct and continuous observation during exposure. We confirmed the method when identifying the sub-lethal concentration range for imidacloprid in one of the species, *H. viatica*.

### Method of dietary exposure to pesticides

Spiking feed by either soaking or moistening for dietary exposure have been applied previously (e.g., Fountain and Hopkin 2001; Tourinho et al. 2015), but not yet compared in ecotoxicological studies. The moistening method provided predictable concentrations, i.e., the measured concentrations were close to the nominal. For the soaked method, nominal concentrations cannot be calculated, as the uptake of imidacloprid in the bark is unknown, challenging the making of predictions for concentrations and subsequent effects. Spiking bark by soaking in a solution concentration we expected to be sub-lethal did result in a too high concentration with consequently high mortality of juveniles of both species. This is consistent with Sengupta et al. (2021), who found high concentrations of imidacloprid in the scraped and filtered Cyanobacteria spiked by soaking overnight. As imidacloprid accumulates in soil with a high content of organic matter (Liu et al. 2006; Knoepp et al. 2012, Zhang et al. 2018), our elevated concentrations in soaked bark are likely explained by a high content of organic carbon in the bark, where imidacloprid can partition. The use of intact bark with a natural layer of Cyanobacteria in the present study resulted in reproduction rates as expected in all three types of control, and was thus was an optimal dietary source for both species. The positive relationship found between moulting and body size further supports the method, reflecting that the monitoring of endpoints is reliable. Also, our two experiments with *H. viatica* produced comparable responses to the same measured dietary exposure concentrations.

### Experiment 1 – Species comparison of survival and sub-lethal responses

The similar survival responses for *H. viatica* and *F. quadrioculata* between treatments in experiment 1 are most likely explained by the difference in exposure concentrations between the two spiking methods, i.e., high exposure from the soaked bark led to high mortality and low exposure from the moistened bark led to low mortality. Exposure to imidacloprid through moistened bark gave high survival, not different from the controls, reflecting that this was a sub-lethal exposure concentration for both species. Neither species reproduced when exposed to soaked bark, likely due to imidacloprid toxicity. Contrastingly, the survival of adult *F. quadrioculata* was not affected by 14 days of exposure to imidacloprid through the diet up to 290 mg/kg dry bark (Sengupta et al. 2021), which is 36 times higher than our treatment with no survival of juveniles (8 mg/kg dry bark, measured in soaked bark) after 20 days. However, the higher susceptibility of the early developmental stages compared with adults (An et al. 2013; Sengupta et al. 2023), explains this difference and underlines the need for understanding tolerance levels in early life stages.

Imidacloprid exposure resulted in 8% reduction in the body sizes of *F. quadrioculata* at 50 days and *H. viatica* at 40 days, when reproductive maturity was expected. However, while the age at first reproduction was not delayed for *F. quadrioculata*, and they reproduced as normal with the smaller body size, only two of five *H. viatica* replicates produced eggs within the experimental time, which could be due to their reduced growth rate. Thus, the two species appear to differ in energy trade-offs between growth and reproduction when exposed to imidacloprid, with *H. viatica* being more sensitive than *F. quadrioculata*. The two species are adapted to different habitats (Hertzberg et al. 2000; Krab et al. 2010; Ponge 2000; Witteveen and Joosse 1987), being exposed to different types and ranges of drivers and stressors in their natural environment, which can affect their tolerance to contaminant stress (Schnug et al. 2014; Konestabo et al. 2022). On the soil surface*, H. viatica* experience rapid changes in temperature, rainfall, and wind, compared to the litter-dwelling *F. quadrioculata*, which is hidden in the soil and more protected from such external factors. The latter species, however, experiences a more complex chemical environment of the soil habitat and pore water. Species differences in energy demand can be another possible explanation as to why *H. viatica* is more sensitive to contaminant stress compared to *F. quadrioculata*. Assimilated energy is divided between production, i.e., growth and reproduction, and respiration, which is maintenance through general metabolism and locomotor activity (Congdon et al. 2001). As *H. viatica* is a more active species compared to *F. quadrioculata* (Hertzberg et al. 2000; Krab et al. 2010), its energy consumption used for maintenance is likely higher, thus leaving less energy available for growth and reproduction. The high mobility of the springtail *Orchesella cincta* caused a higher energy demand for maintenance compared to a Collembola species with lower mobility (*Tomocerus minor*) (Testerink 1982), and reduced growth was hypothesised to be linked to high locomotor activity in *Folsomia candida* (Collembola) (Martikainen and Rentalainen 1999). Moreover, as *H. viatica* is very mobile, it can migrate when suboptimal conditions occur in search of better-quality food or to avoid contaminated food (Jensen et al. 2006). Avoidance of feed contaminated with imidacloprid was suggested for *H. viatica* based on similar internal concentrations in animals exposed to different concentrations of imidacloprid in their feed (Kristiansen et al. 2021). However, reduced consumption also reduces the energy available for maintenance and production. Other possible factors impacting species tolerance to pesticide exposure are differences in physiology, body size, and their capacity for biotransformation of the specific compound. Our findings confirm our assumption on species sensitivity differences and emphasise the variation found within a large animal taxon, such as Collembola. Thus, several species should be studied (e.g., Son et al. 2007; van Gestel 2012) to assess how pesticides affect the soil fauna responses across adaptations and different life-history traits.

### Experiment 2 – Responses to sub-lethal concentrations of imidacloprid

Our imidacloprid concentration range from 0.01 to 1.2 mg/kg dry bark was optimal for studying sub-lethal responses of *H. viatica* with high resolution: all endpoints were unaffected at the lowest concentration, the three intermediate concentrations induced negative sub-lethal effects, and the highest concentration reduced survival. Our three sub-lethal endpoints, age and body size at first reproduction and moulting rate were negatively affected by imidacloprid, but had different thresholds for effect. Body size at first reproduction was the most sensitive trait with LOEC 0.04 mg/kg dry bark, followed by age at first reproduction with LOEC 0.4 mg/kg dry bark. Lastly, the LOEC for moulting rate was 1.2 mg/kg dry bark, thus not affected by the sub-lethal concentrations. Our findings emphasise the importance of studying several life-history traits as a supplement to the traditional endpoints, mortality and juvenile recruitment, as toxic effect thresholds are trait-specific (Jensen et al. 2001; Crouau and Moia 2006; Lee et al. 2019), and will have an overall effect on population dynamics.

We suggest that the egg production per se was not physiologically impaired in *H. viatica*, but rather delayed due to the imidacloprid exposure in our second experiment. The age at first reproduction was higher in animals exposed to the highest sub-lethal concentration of 0.4 mg/kg dry bark, but a delay was also indicated for 0.13 mg/kg dry bark as only four of five replicates reproduced within the experiment duration. This corresponds with experiment 1, of which three of five replicates did not reproduce within the approximate same duration, when exposed to the measured concentration of 0.21 mg/kg dry bark measured imidacloprid in moistened bark. As eggs were produced in some replicates, it is likely that all replicates would eventually reproduce if the experiment duration was prolonged. Similarly, a delay in first reproduction was also found as a response in *F. candida* exposed to the insecticide Trebon, with the active ingredient etofenprox (Szabo et al. 2020). A delay in age at first reproduction is not necessarily the same as a delay in maturation, as the animal physiology may be mature for reproduction, but not reproducing due to absence of suitable environmental cues (Ernsting et al. 1993). *F. candida* fed baker´s yeast reproduced at an earlier age with no compensation in size, compared to those fed less favourable feed, pollen and fungal spores (Stam et al. 1996). Thus, age and body size at first reproduction are not necessarily dependent, and more optimal environmental conditions, here food supply, impact the timing of the first reproductive event (Stam et al. 1996). Similar findings with food type influencing age at first reproduction have been done for several other Collembola species (Hoskins et al. 2015), and the timing of reproduction can therefore be affected by contaminated food indirectly, if the animals can detect contamination and avoid it, causing a reduced energy budget.

Collembola species with indeterminate growth, i.e., increase in body size during their whole life such as *H. viatica*, do not have a fixed body size for first reproduction (Ernsting et al. 1993; Sengupta et al. 2017). A delay in first reproduction can result in increased total fecundity, if higher age leads to a larger body size, and thus larger physiological capacity for producing larger or a higher number of eggs (Ernsting et al. 1993). A trade-off between growth and reproduction could be expected in favour of growth (Dai et al. 2020; Szabo et al. 2020), but in experiment 1 of the present study we found a reduced body size but a normal reproduction rate for *F. quadrioculata*. However, for *H. viatica* a reduced growth rate was found in both experiments, and in the second, body size was the most sensitive trait. Therefore, it is likely that the delay in age at reproduction would correlate with reduced fecundity over time for *H. viatica*. The negative response in both age and size at first reproduction could be due to a smaller energy budget caused by avoidance of contaminated food (Dai et al. 2018; Kristiansen et al. 2021), as a growing body needs increasing amounts of energy for maintenance, as well as for detoxification of imidacloprid.

Contaminant stress can cause a reduction in moulting rate (Lee et al. 2016; Al-Badran et al. 2019; Dai et al. 2020), and we found that imidacloprid reduced moulting by 78% at the lethal concentration (1.2 mg/kg dry bark) with no registered reproduction. However, moulting was unaffected by the sub-lethal concentrations where we registered reproduction. As we saw a positive relationship between body size and moulting, i.e., confirming moulting as a proxy for growth, our results might indicate that the animals were large enough to reproduce, i.e., mature, but that any energy allocation induced to maintain homeostasis was directed towards survival. Moreover, unlike concentrations up to 0.4 mg/kg dry bark, our findings suggest that reproduction would likely not occur at the highest concentration of 1.2 mg/kg dry bark due to high toxicity. A decreased moulting rate reflects highly reduced growth, but also potential reduction in elimination capacity. As Collembola renovate their epithelium as an excretion mechanism during moulting (Joosse and Buker 1979), moulting is a potential route of elimination of anthropogenic contaminants. Thus, a reduction in moulting rate can also potentially increase the toxicity of imidacloprid. Toxicity was reflected in the high mean mortality of 77%, which also entails that there are fewer partners available for reproduction, and therefore reduced contribution to the gene pool. Consequently, when exposed from a juvenile age, exposure to this concentration would likely cause a fast population decline.

In summary, the moistened bark was optimal for dietary exposure to imidacloprid and allowed continuous monitoring and documentation of sub-lethal effects during exposure in two naturally abundant and widely distributed Collembola species. While imidacloprid reduced the growth rate in both *F. quadrioculata* and *H. viatica*, a negative effect on reproduction was only found in *H. viatica*. In the following concentration-response experiment on *H. viatica*, imidacloprid delayed the age and reduced the size at first reproduction as the most sensitive traits. This suggests a trade-off in energy allocation between maintenance and reproduction, which needs further attention in future studies, as a reduction in reproduction can potentially have a large effect on population growth.

### Supplementary information

Supplementary Information
