# Supplementary material for: Using dietary exposure to determine sub-lethal effects from imidacloprid in two springtail (Collembola) species
Source: Ecotoxicology. 2023 Nov 21;32(10):1209–20. doi: 10.1007/s10646-023-02715-x (PMC10724306; doi:10.1007/s10646-023-02715-x)
Supplement: Supplementary file 1 — Supplementary Information [file 10646_2023_2715_MOESM1_ESM.docx]

**Supplementary information for**

Using dietary exposure to determine sub-lethal effects from imidacloprid in two springtail (Collembola) species

Ecotoxicology

Andreia Sofia Jorge Silva^a,b^, Silje Marie Kristiansen^a^, Sagnik Sengupta^a^, Cornelis A. M. van Gestel^c^, Hans Petter Leinaas^a^, Katrine Borgå^a*^

^a^Section for Aquatic Biology and Toxicology, Department of Biosciences, University of Oslo, Norway

^b^Department of Animal Biology, Faculty of Sciences, University of Lisbon, Portugal.

^c^Amsterdam Institute for Life and Environment (A-LIFE), Faculty of Science, Vrije Universiteit, Amsterdam, The Netherlands

*Corresponding author: katrine.borga@ibv.uio.no

Content:

A short supplementary text (S1), and figure results referenced in main text (Figure S1-S5).

*S1: comment on using bark with cyanobacteria as dietary exposure medium*

In our previous studies, we applied the similar bark collected in the same area as in the present study, but the layer of cyanobacteria was scraped off using a scalpel, and then sieved in a mesh to homogenize the powder size, before soaked in an imidacloprid solution overnight and filtered. The controls of *H. viatica* stopped reproducing after some days feeding of the filtered cyanobacteria, indicating a sub-optimal feed (Kristiansen et al., 2021). For *F. quadrioculata*, juvenile individuals were with imidacloprid-induced impaired movements stuck on the feed, making it difficult to replace the feed without harming the juveniles and substantially increasing the handling time for each replicate (Sengupta et al., 2021).

*S2: comment on censoring lost animals with Kaplan-Meier statistics*

Due to high toxicity from the soaked spiked bark in experiment 1, numerous individuals showed reduced development and growth, remaining white like newly hatched animals, rather than developing a darker colour. Several of these individuals with halted development were stuck on the bark, immobilised, which complicated the renewal of bark. In the soaked spiked treatment, animals that were not registered as dead were at some point lost, most likely due to being hard to observe on the bark that was replaced. However, as we were not certain exactly which day most of the animals were lost, they were censored halfway through the experiment, i.e., at day 20 for *F. quadrioculata* and day 25 for *H. viatica*. For experiment 2, the animals were counted at each observation and the censoring was thus more specific compared to experiment 1.

### *Figures:*

### Image of the food source, bark with natural layer of Cyanobacteria:


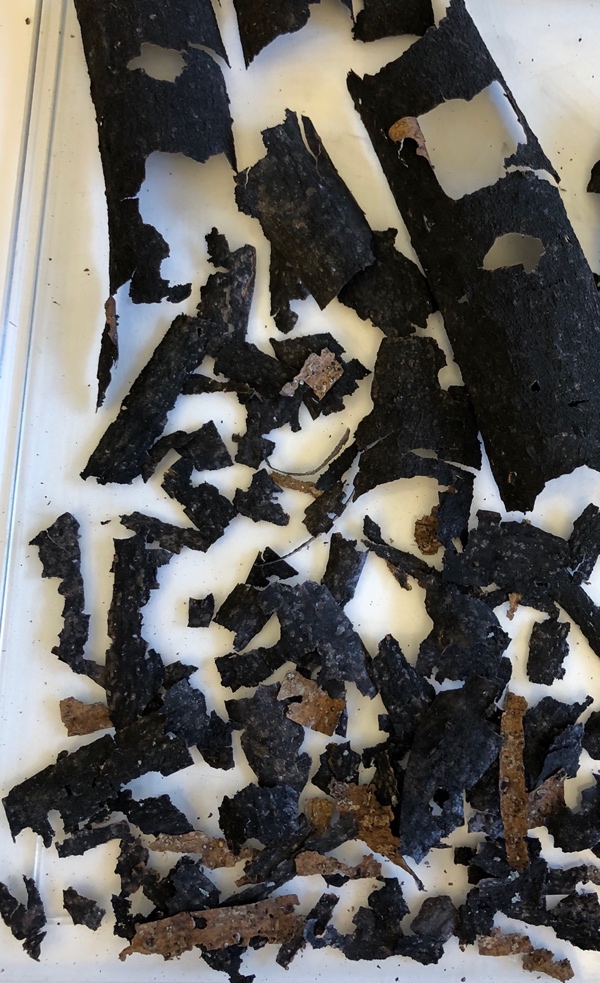


**Fig. S1** Bark with a natural layer of Cyanobacteria, peeled off dead branches from Littleleaf Linden (*Tilia cordata*) trees, Oslo, Norway.

### Age at first reproduction for *Folsomia quadrioculata* and *Hypogastrura viatica:*

**Fig. S2** Age at first reproduction for a) *Folsomia quadrioculata* (Collembola) and b) *Hypogastrura viatica (Collembola)*, exposed dietary to imidacloprid through bark comparing two spiking methods: soaked in an imidacloprid solution overnight versus moistened with a droplet of imidacloprid solution using a micro-pipette. The spiked treatments had a measured imidacloprid concentration of 0.21 mg/kg and 8.0 mg/kg for the moistened and soaked spiking method, respectively. Bark soaked with imidacloprid caused high mortality and no reproduction, hence n=0. Data presented as median, quartiles and 10-90 percentiles

### Adult body size for *Folsomia quadrioculata* and *Hypogastrura viatica:*

**Fig. S3** Body size of a) Folsomia quadrioculata (Collembola) at 40 days of age, and b) Hypogastrura viatica (Collembola) at 50 days of age, dietary exposed to imidacloprid through bark comparing two spiking methods: soaked in an imidacloprid solution overnight versus moistened with a droplet of imidacloprid solution using a micro-pipette. The spiked treatments had a measured imidacloprid concentration of 0.21 mg/kg and 8.0 mg/kg for the moistened and soaked spiking method, respectively. Bark soaked with imidacloprid caused high mortality and no reproduction, hence n=0. Data presented as median, quartiles, 10-90 percentiles and outliers. (**) represents significant level p < 0.001 in comparison with the controls (Tukey HSD test)

Age and body size at first reproduction for *Hypogastrura viatica*:

**Fig. S4** Age and median body size at first reproduction for Hypogastrura viatica (Collembola) dietary exposed to imidacloprid (mg/kg dry bark)

###

Moulting rate for *Hypogastrura viatica*:

**Fig. S5** Moulting rate of *Hypogastrura viatica* (Collembola) dietary exposed to imidacloprid (mg/kg dry bark)

Body size and moulting rate for *Hypogastrura viatica*:


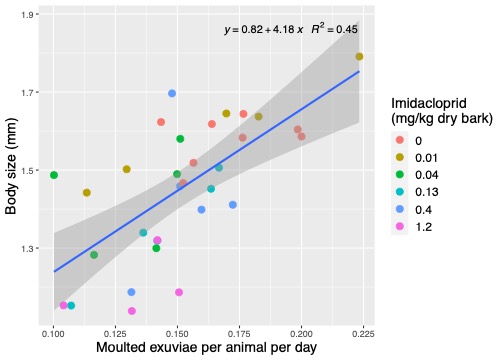


**Fig. S6** Body size and moulting rate of *Hypogastrura viatica* (Collembola), dietary exposed to imidacloprid
